# Spectrum of variants associated with inherited retinal dystrophies in Northeast Mexico

**DOI:** 10.1186/s12886-023-03276-7

**Published:** 2024-02-12

**Authors:** Rocio A. Villafuerte-de la Cruz, Lucas A. Garza-Garza, Manuel Garza-Leon, Cesar Rodriguez-De la Torre, Cinthya Parra-Bernal, Ilse Vazquez-Camas, David Ramos-Gonzalez, Andrea Rangel-Padilla, Angelina Espino Barros-Palau, Jose Nava-García, Javier Castillo-Velazquez, Erick Castillo-De Leon, Agustin Del Valle-Penella, Jorge E. Valdez-Garcia, Augusto Rojas-Martinez

**Affiliations:** 1 Tecnologico de Monterrey, Escuela de Medicina y Ciencias de La Salud, Ave. Morones Prieto 3000, Col. Los Doctores, Monterrey, CP 64710 Mexico; 2 Destellos de Luz, San Pedro Garza García, México; 3 Clinical Science Department, Health Sciences Division, University of Monterrey, Monterrey, México; 4 Tecnologico de Monterrey, The Institute for Obesity Research, Ave. Morones Prieto 3000, Col. Los Doctores, Monterrey, CP 64710 Mexico; 5 Instituto de La Visión, Hospital La Carlota, Montemorelos, México; 6 Oftalmolaser, Monterrey, México; 7 Tecnologico Nacional de Mexico Campus Tuxtla Gutierrez, Tuxtla Gutierrez, Mexico

**Keywords:** Retinal dystrophies, Inherited retinopathy, Pathogenic variant, Mexico

## Abstract

**Background:**

Inherited retinal dystrophies are hereditary diseases which have in common the progressive degeneration of photoreceptors. They are a group of diseases with clinical, genetic, and allelic heterogeneity. There is limited information regarding the genetic landscape of inherited retinal diseases in Mexico, therefore, the present study was conducted in the northeast region of the country.

**Methods:**

Patients with inherited retinal dystrophies were included. A complete history, full ophthalmological and medical genetics evaluations, and genetic analysis through a targeted NGS panel for inherited retinal dystrophies comprising at least 293 genes were undertaken.

**Results:**

A total of 126 patients were included. Cases were solved in 74.6% of the study’s population. Retinitis pigmentosa accounted for the most found inherited retinal disease. Ninety-nine causal variants were found, being *USH2A* and *ABCA4* the most affected genes (26 and 15 cases, respectively).

**Conclusions:**

The present study documents the most prevalent causative genes in IRDs, as *USH2A*, in northeastern Mexico. This contrasts with previous reports of IRDs in other zones of the country. Further studies, targeting previously unstudied populations in Mexico are important to document the genetic background of inherited retinal dystrophies in the country.

**Supplementary Information:**

The online version contains supplementary material available at 10.1186/s12886-023-03276-7.

## Introduction

Inherited retinal dystrophies (IRDs) are characterized by progressive degeneration of photoreceptors, resulting in vision loss that may develop from birth to late middle age [1]. IRDs comprise a variety of overlapping conditions, including retinitis pigmentosa (RP), Stargardt disease/macular dystrophy (STGD/MD), cone-rod dystrophies (CRD), Leber congenital amaurosis (LCA) and syndromic forms such as Usher syndrome. Collectively, they have a prevalence of ~ 1 in 2,000–3,000 people [2, 3] and are estimated to affect up to 5.5 million individuals worldwide [4]. Rod dominant dystrophies, such as RP, present with peripheral vision loss and night blindness [5]. By contrast, cone dominant dystrophies, such as STGD/MD and CRD, present with central vision loss and impaired color perception, photophobia, and nystagmus [5, 6]. As both types of dystrophies progress, rod and cones may undergo degeneration compromising both central and peripheral vision at end stages. LCA is the most severe type of IRD, affecting both photoreceptors and the retinal pigment epithelium, with symptoms appearing during the first year of life [6].

IRDs exhibit both genetic and clinical heterogeneity. All inheritance patterns have been reported among IRDs, including autosomal, X-linked, mitochondrial, or digenic patterns [7, 8]. Currently, more than 200 causative genes have been identified, with the majority being autosomal recessive conditions [7, 8]. IRDs show considerable genetic and allelic heterogeneity [2, 3]. For example, *ABCA4* mutations have been associated with the development of STGD, RP, CRD, and age-related macular degeneration [9]. Furthermore, intrafamilial variability is common among RDs [10, 11] and it is partly explained by environmental or genetic modifiers, specifically, mutations in other IRDs genes or single nucleotide variants [3].

Identification of the causative genetic variants is essential to ensure an accurate diagnosis and to provide a reference for genetic counseling [8]. In addition, understanding the molecular mechanism of IRDs is leading to the development of therapeutic interventions that seek to halt the loss of photoreceptors and vision preservation [3, 12]. Several studies have employed next-generation sequencing (NGS) techniques in multiple cohorts of RDs patients, with detection rates of molecular defects in ~ 60% of cases [6, 7]. The overall detection rate is not as high as expected for several reasons, including, but not limited to, variants in intronic sequences, uncharacterized genes, variants affecting mRNA splicing, and structural variants, such as copy number variations, duplications, or inversions [5, 7, 8]. There is a large variability of genes and mutations causing IRDs among different populations, and molecular analysis of understudied groups will allow for the reclassification of variants of unknown significance into pathogenic variants [4, 7]. Currently, there is limited data on the underlying genetic variants in families of Mexican descent. Furthermore, the available research has focused on IRDs patients from central and south Mexico [13, 14]. Therefore, the present study was undertaken to contribute to this growing area of research by analyzing the mutation spectrum of IRDs-associated genes in Northeastern Mexican patients, i.e., the states of Coahuila, Nuevo Leon, and Tamaulipas.

## Methods

The study protocol was approved by the Institutional Review Board of the School of Medicine at Tecnologico de Monterrey (code P000625-DIMDRET-CEIC-CR001), and all procedures were conducted in compliance with the Declaration of Helsinki. Written informed consent was obtained from all the patients or their legal guardians.

The study population comprised 126 unrelated patients who were selected based on: (1) IRD diagnosis, (2) origin/residence in Northeastern Mexico (Coahuila, Nuevo Leon, and Tamaulipas), and (3) grandparents born in Mexico. Participants were recruited in the following outpatient clinics: Fundación Santos y de la Garza Evia, Fundación Destellos de Luz, Instituto de la Visión of Hospital La Carlota, and from the private practice. IRD diagnosis was based on clinical examination, including uncorrected and corrected visual acuity, fundus examination, visual field testing, fundus autofluorescence, and spectral-domain optical coherence tomography scan. Full-field electroretinography was performed when available. A clinical geneticist collected demographic and familiar data, including family pedigree, age of onset of symptoms, and presence of systemic findings.

DNA sample was extracted from saliva or buccal swab and analyzed with a targeted NGS panel for inherited retinal dystrophies comprising at least 293 genes at Invitae Corp. (San Francisco, CA). Targeted regions were enriched using a hybridization-based protocol and sequenced using Illumina technology. Exon deletions and duplications were assessed using an internal algorithm that compared read-depth for each target sequence in the proband to internal control samples. Classification of variants was based on the American College of Medical Genetics and Genomics (ACMG) guidelines.

## Results

Genetic testing was performed on a total of 126 probands with 74 females and 52 males. Probands were natives/residents of Nuevo Leon (94), Tamaulipas (20) and Coahuila (12). The average age of the probands at the time of testing was 39.06 ± 18.64 years (range 4–82 years). The median age at symptoms onset was 13 years (IQ range 17.5) (range 2 months to 70 years). The full demographic and clinical data of the patients is shown in Supplementary material Table 1.

The initial diagnoses in this cohort, according to clinical presentation and examination (Table 2), were: Non-syndromic IRD: RP (53 cases), STGD/MD (21 cases), CRD (15 cases), LCA (3 cases), X-linked retinoschisis (4 cases). Syndromic IRD: Usher 2A syndrome (25 cases), Bardet Biedl syndrome (2 cases), Alstrom syndrome (1 case), 1 case with intellectual disability, short stature, deafness, optic atrophy, and RP, and 1 case with intellectual disability, deafness, coarse facies, and late onset RP.

Cases were classified as solved, partially solved, and unsolved according to a previous work [13] (Table 1). The causative variant detection rate (solved cases) in this cohort was 74.6% (94/126) (Table 2). Partially solved cases were detected in 10/126. A total of 22/126 cases remained unsolved.

**Table 1:** Case classification

**Solved cases**
** Autosomal dominant**
Pathogenic or likely pathogenic variant in heterozygous state
** Autosomal recessive**
Pathogenic or likely pathogenic variant in homozygous or compound heterozygous state
Pathogenic or likely pathogenic variant in heterozygous state and a VUS in the other AR allele, plus clinical correlation
** X-linked**
Pathogenic or likely pathogenic variant in hemizygous or heterozygous state, plus clinical correlation
**Partially solved**
Pathogenic or likely pathogenic variant in heterozygous state in an AR allele, plus clinical correlation
**Suspected causal VUS**
VUS with clinical correlation
**Unsolved**
No pathogenic or likely pathogenic variants. VUS without clinical correlation

**Table 2:** Comparison of the three available cohorts from IRDs sequencing in Mexico

	**Villanueva-Mendoza et al **[13]	**Zenteno et al [**14]	**Present study**
***n***	**144 (100%)**	**143 (100%)**	**126 (100%)**
***Pre Sequencing diagnosis***			
**RP**	**47 (32.6%)**	**85 (52.4%)**	**53 (42.1%)**
**LCA and EORD**	**33 (22.9%)**	**21 (14.6%)**	**3 (2.4%)**
**Other IRDs**	**37 (25.7%)**	**18 (12.5%)**	**40 (31.7%)**
**Syndromic IRDs**	**20 (13.9%)**	**19 (13.2%)**	**30 (23.8%)**
**Causative variant detection**	**105 (72.9%)**	**95 (66%)**	**94 (74.6%)**
***Variations classification***			
**Missense**	**52.7%**	**49%**	**47%**
**Frameshift**	**21.3%**	**25%**	**16%**
**Nonsense**	**10.0%**	**15%**	**14%**
**Splicing**	**7.3%**	**7%**	**11%**
**Others**	**8.6%**	**4%**	**11%**
**Most commonly affected gene**	***ABCA4*** **19 (18%)**	***ABCA4*** **8 (8%)**	***USH2A*** **27 (27%)**
**Unsolved cases**	**22 (15%)**	**48 (34%)**	**22 (17%)**

### Molecular findings / genetic profile of IRD patients

Total different causative variants were 99 among 37 genes, including 96 single nucleotide variants (SNVs) and 3 copy number variations (CNVs), with a total of 175 alleles (Table 3). According to the ACMG guidelines 82 variants were pathogenic, 9 variants were likely pathogenic, and 8 variants were of uncertain significance (VUS). The phenotype and genotype data of the present study were deposited in the LOVD database v.3.0 [15]. Most of the variants were compound heterozygous (49 cases), followed by homozygous (24 cases), heterozygous (13 cases), and hemizygous (8 cases). Most of the variants were determined to be compound heterozygote in 49 cases, followed by homozygous in 24, heterozygous in 13 and hemizygous in 8. DNA changes were predominately missense variants 47, followed by frameshift 16, nonsense 14, splicing 11, intronic 4, CNVs 3, InFrame Indel 2, synonymous 1, start loss 1 (Table 2).

**Table 3:** Variants found in the present study

***ID***	**Gene**	**NM ID**	**Zygocity**	**cDNA change**	**Protein change**	**ACMG**	**Reference**
***ar RP***							
*24*	*CABP4*	NM_145200.3	Hom	c.154C>T	p.Arg52*	PV	[16]
*35*	*CEP78*	NM_001098802.1	Hom	c.473G>T	p.Cys158Phe	LPV	Novel
*2*	*CLN3*	NM_001042432.1	Het	c.944dup	p.His315Glnfs*67	PV	[17]
*2*	*CLN3*	NM_001042432.1	Het	c.1305C>G	p.Cys435Trp	VUS	Novel
*13*	*CLN3*	NM_001042432.1	Het	c.1A>G	p.Met1?	PV	[18]
*13*	*CLN3*	NM_001042432.1	Het	c.464T>G	p.Val155Gly	VUS	Novel
*39*	*CNGA1*	NM_000087.3	Het	c.652C>T	p.Arg218*	PV	[19]
*39*	*CNGA1*	NM_000087.3	Het	c.1065G>C	p.Trp355Cys	VUS	Novel
*85*	*CNGB1*	NM_001297.4	Hom	c.290+2T>C Splice donor		LPV	[20]
*92*	*CNGB1*	NM_001297.4	Hom	c.2957A>T	p.Asn986Ile	PV	[21]
*84*	*CRB1*	NM_201253.2	Het	c.2290C>T	p.Arg764Cys	PV	[22]
*84*	*CRB1*	NM_201253.2	Het	c.2171_2172del	p.Tyr724Cysfs*6	PV	[23]
*10*	*EYS*	NM_001142800.1	Het	c.4120C>T	p.Arg1374*	PV	[24]
*10*	*EYS*	NM_001142800.1	Het	c.6079-2A>G Splice acceptor		LPV	Novel
*90*	*EYS*	NM_001142800.1	Het	c.5928-2A>G Splice acceptor		PV	[20]
*90*	*EYS*	NM_001142800.1	Het	c.6794del	p.Pro2265Glnfs*46	PV	[25]
*63*	*IFT172*	NM_015662.2	Het	c.4868C>T p.Thr1623Ile	p.Thr1623Ile	LPV	Novel
*63*	*IFT172*	NM_015662.2	Het	c.4876_4878dup	p.Pro1626dup	VUS	Novel
*56*	*KIZ*	NM_018474.4	Hom	c.226C>T	p.Arg76*	PV	[26]
*48*	*PDE6A*	NM_000440.2	Het	c.1705C>A	p.Gln569Lys	PV	[27]
*48*	*PDE6A*	NM_000440.2	Het	c.1957C>T	p.Arg653*	PV	[28]
*94*	*PDE6A*	NM_000440.2	Hom	c.2053G>A	p.Val685Met	PV	[29]
*18*	*USH2A*	NM_206933.2	Het	c.2276G>T	p.Cys759Phe	PV	[30]
*18*	*USH2A*	NM_206933.2	Het	c.2299del	p.Glu767Serfs*21	PV	[31]
*50*	*USH2A*	NM_206933.2	Hom	c.2276G>T	p.Cys759Phe	PV	[30]
*68*	*USH2A*	NM_206933.2	Het	c.2276G>T	p.Cys759Phe	PV	[30]
*68*	*USH2A*	NM_206933.2	Het	c.2299del	p.Glu767Serfs*21	PV	[31]
*83*	*USH2A*	NM_206933.2	Het	c.2276G>T	p.Cys759Phe	PV	[30]
*83*	*USH2A*	NM_206933.2	Het	c.2299del	p.Glu767Serfs*21	PV	[31]
*88*	*USH2A*	NM_206933.2	Het	c.10820A>C	p.His3607Pro	PV	[32]
*88*	*USH2A*	NM_206933.2	Het	c.12575G>A	p.Arg4192His	PV	[33]
*93*	*USH2A*	NM_206933.2	Het	c.12067-2A>G Splice acceptor		LPV	[31]
*93*	*USH2A*	NM_206933.2	Het	c.8188C>A	p.Pro2730Thr	VUS	Novel
***ad RP***							
*14*	*IMPDH1*	NM_000883.3	Het	c.931G>A	p.Asp311Asn	PV	[32]
*77*	*PRPF3*	NM_004698.2	Het	c.1477C>T (p.Pro493Ser)		PV	[34]
*80*	*PRPH2*	NM_000322.4	Het	c.514C>T	p.Arg172Trp	PV	[35]
*19*	*SAG*	NM_000541.4	Het	c.440G>T	p.Cys147Phe	PV	[34]
*45*	*SAG*	NM_000541.4	Het	c.440G>T	p.Cys147Phe	PV	[34]
*51*	*SAG*	NM_000541.4	Het	c.440G>T	p.Cys147Phe	PV	[34]
*57*	*SNRNP200*	NM_014014.4	Het	c.2580G>C	p.Gln860His	PV	Novel
***xl RP***							
*64*	*RPGR*	NM_001034853.2	Hem	c.2426_2427del	p.Glu809Glyfs*25	PV	[36]
*66*	*RPGR*	NM_000328.2	Hem	Deletion Exons 8-18		PV	Novel
*69*	*RPGR*	NM_001034853.2	Het	c.1206_1215del	p.Gln403Tyrfs*19	PV	[37]
*78*	*RPGR*	NM_000328.2	Hem	c.934+1G>C Splice donor		PV	[38]
*28*	*RP2*	NM_006915.2	Hem	c.542_543del	p.Ser181Trpfs*37	PV	[39]
*86*	*RP2*	NM_006915.2	Hem	c.102G>A Silent		LPV	[40]
***CRD***							
*49*	*CFAP410*	NM_004928.2	Het	c.347C>T	p.Pro116Leu	PV	[41]
*49*	*CFAP410*	NM_004928.2	Het	c.115_117dup	p.Met39dup	VUS	Novel
*72*	*CNGA3*	NM_001298.2	Het	c.1228C>T	p.Arg410Trp	PV	[42]
*72*	*CNGA3*	NM_001298.2	Het	c.1585G>A	p.Val529Met	PV	[42]
*82*	*CNGB3*	NM_019098.4	Het	c.1810C>T	p.Arg604*	PV	[43]
*82*	*CNGB3*	NM_019098.4	Het	c.701_702delinsAG	p.Cys234*	PV	[44]
*79*	*PDE6C*	NM_006204.3	Hom	c.221del	p.Gly74Alafs*69	PV	[45]
*12*	*POC1B*	NM_172240.2	Hom	c.144del	p.Lys48Asnfs*16	PV	[46]
*71*	*POC1B*	NM_172240.2	Het	c.676+1G>A (Splice donor)		PV	[20]
*71*	*POC1B*	NM_172240.2	Het	c.320G>T	p.Ser107Ile	VUS	Novel
*9*	*PROM1*	NM_006017.2	Het	c.2130+2del (Splice site)		PV	[47]
*9*	*PROM1*	NM_006017.2	Het	c.1423_1424del	p.Val475Leufs*42	PV	[48]
*16*	*PROM1*	NM_006017.2	Hom	c.2130+2del (Splice site)		PV	[47]
*7*	*USH2A*	NM_206933.2	Het	c.2276G>T	p.Cys759Phe	PV	[30]
*7*	*USH2A*	NM_206933.2	Het	c.9799T>C	p.Cys3267Arg	PV	[49]
***STGD/ MD***							
*5*	*ABCA4*	NM_000350.2	Hom	c.4926C>G	p.Ser1642Arg	PV	[49]
*5*	*ABCA4*	NM_000350.2	Hom	c.5044_5058del	p.Val1682_Val1686del	PV	[50]
*6*	*ABCA4*	NM_000350.2	Het	c.5318C>T	p.Ala1773Val	PV	[51]
*6*	*ABCA4*	NM_000350.2	Het	c.634C>T	p.Arg212Cys	PV	[52]
*15*	*ABCA4*	NM_000350.2	Het	c.1804C>T	p.Arg602Trp	PV	[53]
*15*	*ABCA4*	NM_000350.2	Het	c.3386G>T	p.Arg1129Leu	PV	[50]
*22*	*ABCA4*	NM_000350.2	Het	c.2908del	p.Thr970Profs*7	PV	[54]
*22*	*ABCA4*	NM_000350.2	Het	c.5882G>A	p.Gly1961Glu	PV	[55]
*23*	*ABCA4*	NM_000350.2	Het	c.4926C>G	p.Ser1642Arg	PV	[49]
*23*	*ABCA4*	NM_000350.2	Het	c.5044_5058del	p.Val1682_Val1686del	PV	[50]
*23*	*ABCA4*	NM_000350.2	Het	c.5318C>T	p.Ala1773Val	PV	[51]
*27*	*ABCA4*	NM_000350.2	Het	c.3386G>T	p.Arg1129Leu	PV	[50]
*27*	*ABCA4*	NM_000350.2	Het	c.4457C>T	p.Pro1486Leu	PV	[50]
*32*	*ABCA4*	NM_000350.2	Het	c.3386G>T	p.Arg1129Leu	PV	[50]
*32*	*ABCA4*	NM_000350.2	Het	c.6718A>G	p.Thr2240Ala	PV	[56]
*32*	*ABCA4*	NM_000350.2	Het	c.4352+61G>A (Intronic)		LPV	[57]
*41*	*ABCA4*	NM_000350.2	Het	c.4537dup	p.Gln1513Profs*42	PV	[58]
*41*	*ABCA4*	NM_000350.2	Het	c.5461-1G>T (Splice acceptor)		PV	[59]
*44*	*ABCA4*	NM_000350.2	Het	c.3386G>T	p.Arg1129Leu	PV	[50]
*44*	*ABCA4*	NM_000350.2	Het	c.4139C>T	p.Pro1380Leu	PV	[60]
*58*	*ABCA4*	NM_000350.2	Het	c.2894A>G	p.Asn965Ser	PV	[61]
*58*	*ABCA4*	NM_000350.2	Het	c.5196+1137G>A (Intronic)		PV	[62]
*60*	*ABCA4*	NM_000350.2	Het	c.5318C>T	p.Ala1773Val	PV	[51]
*60*	*ABCA4*	NM_000350.2	Het	c.6221G>T	p.Gly2074Val	PV	[51]
*61*	*ABCA4*	NM_000350.2	Het	c.1804C>T	p.Arg602Trp	PV	[53]
*61*	*ABCA4*	NM_000350.2	Het	c.4253+4C>T (Intronic)		PV	[63]
*62*	*ABCA4*	NM_000350.2	Het	c.3322C>T	p.Arg1108Cys	PV	[64]
*62*	*ABCA4*	NM_000350.2	Het	c.4139C>T	p.Pro1380Leu	PV	[58]
*81*	*ABCA4*	NM_000350.2	Het	c.3113C>T	p.Ala1038Val	PV	[65]
*81*	*ABCA4*	NM_000350.2	Het	c.6221G>T	p.Gly2074Val	PV	[51]
*91*	*ABCA4*	NM_000350.2	Het	c.4926C>G	p.Ser1642Arg	PV	[49]
*91*	*ABCA4*	NM_000350.2	Het	c.5044_5058del	p.Val1682_Val1686del	PV	[50]
*91*	*ABCA4*	NM_000350.2	Het	c.6581del	p.Pro2194Glnfs*53	PV	[66]
*52*	*ARL3*	NM_004311.3	Het	c.445C>T	p.Arg149Cys	PV	[67]
*3*	*PROM1*	NM_006017.2	Het	c.2130+2del (Splice site)		PV	[47]
*3*	*PROM1*	NM_006017.2	Het	c.220+1G>C (Splice donor)		PV	[68]
*37*	*PROM1*	NM_006017.2	Het	c.2130+2del (Splice site)		PV	[48]
*37*	*PROM1*	NM_006017.2	Het	c.436C>T	p.Arg146*	PV	[48]
*43*	*PROM1*	NM_006017.2	Hom	c.1423_1424del	p.Val475Leufs*42	PV	[48]
*67*	*BEST1*	NM_004183.3	Het	c.851A>G	p.Tyr284Cys	PV	[72]
***LCA***							
*1*	*NMNAT1*	NM_022787.3	Het	c.507G>A	p.Trp169*	PV	[69]
*1*	*NMNAT1*	NM_022787.3	Het	c.769G>A	p.Glu257Lys	PV	[69]
*75*	*CEP290*	NM_025114.3	Het	Gain (Exons 16-26)		PV	Novel
*75*	*CEP290*	NM_025114.3	Het	c.4651C>T	p.Gln1551*	PV	[70]
***RS1***							
*8*	*RS1*	NM_000330.3	Hem	c.208G>A	p.Gly70Ser	PV	[71]
*11*	*RS1*	NM_000330.3	Hem	c.208G>A	p.Gly70Ser	PV	[71]
*89*	*RS1*	NM_000330.3	Hem	c.208G>A	p.Gly70Ser	PV	[71]
***Usher***							
*17*	*ADGRV1*	NM_032119.3	Het	c.10054-2A>C (Splice acceptor)		PV	[73]
*17*	*ADGRV1*	NM_032119.3	Het	c.1563dup	p.Pro522Serfs*8	PV	[73]
*4*	*USH2A*	NM_206933.2	Het	c.1000C>T	p.Arg334Trp	PV	[74]
*4*	*USH2A*	NM_206933.2	Het	c.2299del	p.Glu767Serfs*21	PV	[31]
*20*	*USH2A*	NM_206933.2	Hom	c.486-14G>A (Intronic)		PV	[75]
*25*	*USH2A*	NM_206933.2	Het	c.12067-2A>G Splice acceptor		LPV	[31]
*25*	*USH2A*	NM_206933.2	Het	c.956G>A	p.Cys319Tyr	PV	[76]
*26*	*USH2A*	NM_206933.2	Hom	c.2299del	p.Glu767Serfs*21	PV	[31]
*29*	*USH2A*	NM_206933.2	Het	c.2299del	p.Glu767Serfs*21	PV	[31]
*29*	*USH2A*	NM_206933.2	Het	c.4016T>G	p.Val1339Gly	LPV	[77]
*30*	*USH2A*	NM_206933.2	Hom	c.5278del	p.Asp1760Metfs*10		[78]
*31*	*USH2A*	NM_206933.2	Hom	c.2299del	p.Glu767Serfs*21	PV	[31]
*36*	*USH2A*	NM_206933.2	Hom	c.2299del	p.Glu767Serfs*21	PV	[31]
*38*	*USH2A*	NM_206933.2	Het	c.2276G>T	p.Cys759Phe	PV	[30]
*38*	*USH2A*	NM_206933.2	Het	c.2299del	p.Glu767Serfs*21	PV	[31]
*40*	*USH2A*	NM_206933.2	Hom	c.2299del	p.Glu767Serfs*21	PV	[31]
*46*	*USH2A*	NM_206933.2	Het	c.2299del	p.Glu767Serfs*21	PV	[31]
*46*	*USH2A*	NM_206933.2	Het	c.9424G>T	p.Gly3142*	PV	[31]
*47*	*USH2A*	NM_206933.2	Het	c.1606T>C (p.Cys536Arg)		PV	[79]
*47*	*USH2A*	NM_206933.2	Het	c.2299del	p.Glu767Serfs*21	PV	[31]
*53*	*USH2A*	NM_206933.2	Het	c.2299del	p.Glu767Serfs*21	PV	[31]
*53*	*USH2A*	NM_206933.2	Het	c.956G>A	p.Cys319Tyr	PV	[31]
*55*	*USH2A*	NM_206933.2	Hom	c.12067-2A>G Splice acceptor		LPV	[31]
*59*	*USH2A*	NM_206933.2	Het	c.12067-2A>G Splice acceptor		LPV	[31]
*59*	*USH2A*	NM_206933.2	Het	c.2299del	p.Glu767Serfs*21	PV	[31]
*65*	*USH2A*	NM_206933.2	Het	c.12067-2A>G Splice acceptor		LPV	[31]
*65*	*USH2A*	NM_206933.2	Het	c.956G>A	p.Cys319Tyr	PV	[76]
*70*	*USH2A*	NM_206933.2	Hom	c.2299del	p.Glu767Serfs*21	PV	[31]
*73*	*USH2A*	NM_206933.2	Het	c.2299del	p.Glu767Serfs*21	PV	[31]
*73*	*USH2A*	NM_206933.2	Het	c.12067-2A>G Splice acceptor		LPV	[31]
*74*	*USH2A*	NM_206933.2	Hom	c.2299del	p.Glu767Serfs*21	PV	[31]
*87*	*USH2A*	NM_206933.2	Hom	c.2299del	p.Glu767Serfs*21	PV	[31]
*5*	*USH2A*	NM_206933.2	Hom	c.2276G>T	p.Cys759Phe	PV	[30]
***Other syndromes***							
*76*	*ALMS1*	NM_015120.4	Het	c.10975C>T (p.Arg3659*)		PV	[80]
*76*	*ALMS1*	NM_015120.4	Het	c.1730C>G (p.Ser577*)		PV	[81]
*34*	*ARL6*	NM_177976.2	Hom	c.228C>G (p.Tyr76*)		PV	[14]
*21*	*BBS5*	NM_152384.2	Hom	c.143-1G>C (Splice acceptor)		PV	[82]
*33*	*HGSNAT*	NM_152419.2	Het	Deletion (Exons 1-2)		PV	Novel
*33*	*HGSNAT*	NM_152419.2	Het	c.185T>C (p.Leu62Pro)		VUS	Novel
*54*	*PRPS1*	NM_002764.3	Het	c.250C>T (p.Arg84Trp)		PV	[83]
*42*	*WFS1*	NM_006005.3	Het	c.2189G>A (p.Trp730*)		PV	[84]

*PV* pathogenic variant, *LPV* likely pathogenic variant, *VUS* variant of uncertain significance

Solved cases were classified into non-syndromic IRD 67 and syndromic IRD 27. The most prevalent diagnosis in the non-syndromic solved case was RP, identified in 33 probands, and for syndromic cases was Usher syndrome type 2A found in 21 cases.

There were 58 sporadic cases, the remaining 36 had familial history. Endogamy or consanguinity was documented in 19 and 1 of the cases, respectively. Inheritance pattern was determined as autosomal recessive (AR) in 74 probands, autosomal dominant (AD) in 10 and X-linked (XL) in 10 cases.

**Fig. 1:**
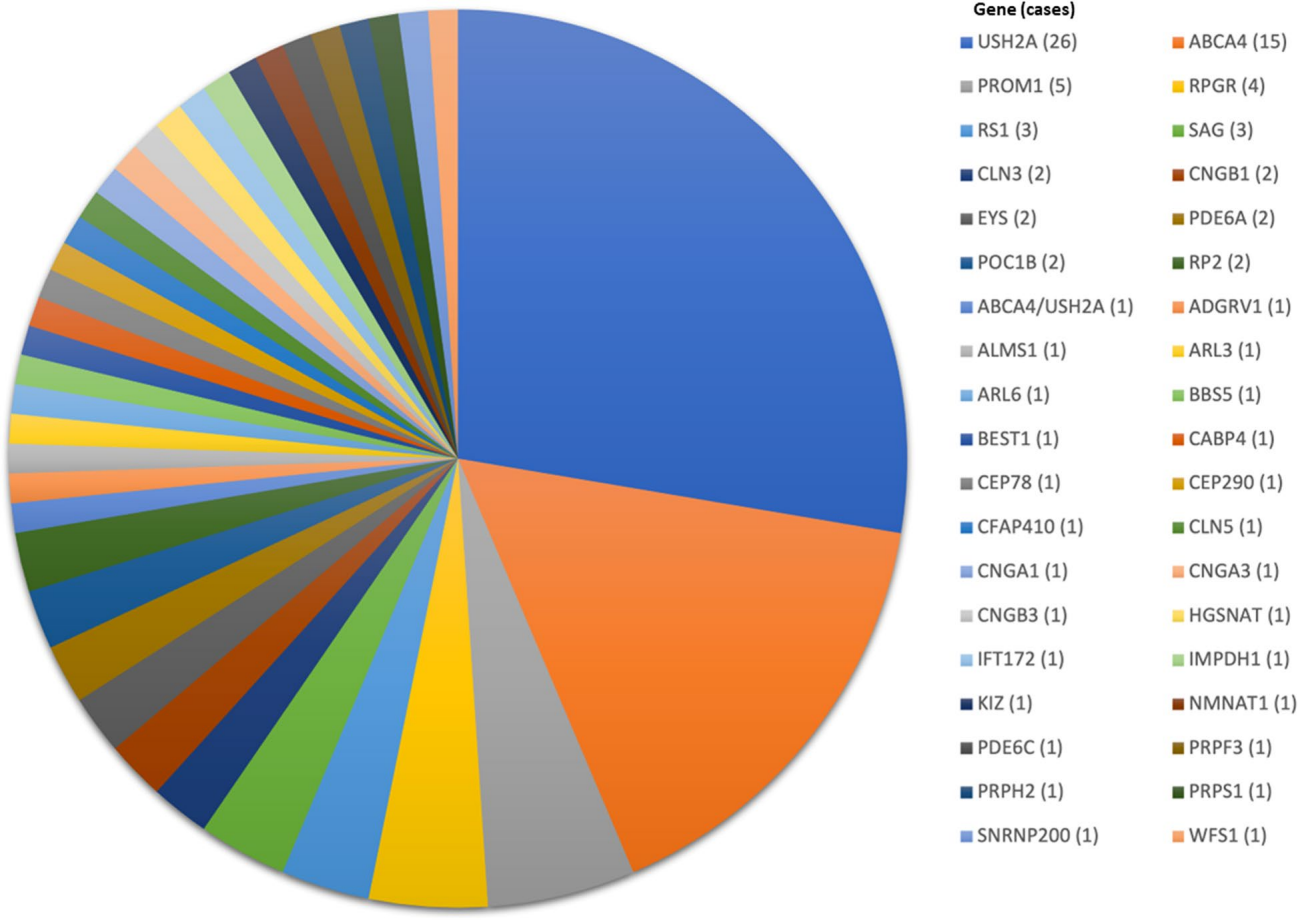
Numbers of cases (in parenthesis) with diagnosed causative genes encountered in the present study

The most frequently causative genes in the solved cases were *USH2A* in 26 and *ABCA4* in 15 (Table 2). The remaining affected genes were *PROM1* in 5, *RPGR* in 4, and *RS1* in 3, and *SAG* in 3, which collectively explain over half of the cases. The remaining genes were represented in 32.9% of the solved cases (Fig. 1).

### Molecular findings in non-syndromic IRD

#### RP findings

RP was the most frequent diagnosis in our cohort. A total of 33 probands were characterized by variants identified in at least one of 18 genes. The diagnostic yield /detection rate was 33/53 cases (62.26%). Seven cases were partially solved, but 13 remain unsolved. Inheritance pattern was determined as autosomal recessive in 20 probands, autosomal dominant in 7, X-linked in 6. The most prevalent affected genes were: AR *USH2A* (6/33), XL *RPGR* (4/33) and, AD *SAG* (3/33). Novel variants were 9, identified in 7 genes: *CLN3* c.1305C > G (p.Cys435Trp) and c.464 T > G (p.Val155Gly), *IFT172* c.4868C > T (p.Thr1623Ile) and c.4876_4878dup (p.Pro1626dup). *CEP78* c.473G > T (p.Cys158Phe). *CNGA1* c.1065G > C (p.Trp355Cys). *EYS* c.6079-2A > G (Splice acceptor). *SNRNP200* c.2580G > C (p.Gln860His) *RPGR* Deletion (Exons 8–18). *USH2A* c.8188C > A (p.Pro2730Thr).

#### CRD findings

In the group of CRD, 15 cases were evaluated. A total of 9 cases were solved. The diagnostic yield/detection rate was 60%. The most common causative genes were *PROM1* and *POC1B* with 2 cases each. The only inheritance mode was AR. One patient was partially solved and 5 remain unsolved. Two novel variants were identified, one in *CFAP410* c.115_117dup (p.Met39dup) and the other in *POC1B* c.320G > T (p.Ser107Ile).

#### STGD/MD findings

For STGD/MD, a total of 21 probands were identified with variants distributed among three genes. The diagnostic yield/detection rate was 20/21 (95%). Most of the cases accounted for autosomal recessive STGD (17/21) due to biallelic variants in *ABCA4* in 14 cases and 3 cases in *PROM1*. The remaining 3 cases corresponded to autosomal dominant in two cases (*BEST1* and *ARL3*) and one case with chromosome 1 isodisomy (*ABCA4*, and *USH2A*). No novel variants were identified in this group.

#### XLR and LCA findings

The remaining non-syndromic diagnoses were distributed as follows: XLR (3/4 cases; *RS1*), LCA (2/3 cases; *CEP290*, and *NMNAT1*). The diagnostic yield/detection rates were 75%, 66.7% respectively. A novel CNV variant was identified in *CEP290* Gain (Exons 16–26) in a patient with LCA.

### Molecular findings in syndromic-IRD

A total of 27/30 cases were solved for this cohort (detection rate:90%), including twenty patients with Usher type 2A syndrome and one Usher type 2C syndrome *(ADGRV1).* Other syndromic diagnoses in this cohort were Bardet-Biedl syndrome (2 cases; *BBS5* and *ARL6*), Alstrom syndrome (1 case; *ALMS1*), Mucopolysaccharidosis type IIIC/ Sanfilippo C (1 case; *HGSNAT*), Arts syndrome (1 case, *PRPS1*), and Wolfram-like syndrome (1 case; *WFS1*). Two novel variants were reported for this group, both in *HGSNAT*: a CNV deletion (Exons 1–2) and a c.185 T > C (p.Leu62Pro) missense variant.

### *USH2A* gene variants

The number of cases associated with *USH2A* is remarkably abundant in this cohort, because 27 patients had causative variants in this gene. Twenty cases were syndromic, six were non-syndromic RP cases whereas one had CRD diagnosis. *USH2A* was the most prevalent affected gene for the whole cohort, with a total of 56 alleles, distributed in 14 variants. For the syndromic phenotype the whole number of alleles were 42, distributed in 10 different variants. The total alleles for non-syndromic RP cases were 12, distributed in 5 variants and only 2 variants for the CRD. The diagnostic yield/detection rate for syndromic Usher 2A was 86.9% (20/23). Family history was reported only in 10/20 of the syndromic cases. The most prevalent pathogenic variants detected in syndromic *USH2A* cases included a frameshift mutation due to c.2299del (p.Glu767Serfs*21) (22/42 alleles; 52.38%), followed by the splicing change c.12067-2A > G (6/42 alleles; 14.28%), and the missense variant c.956G > A (p.Cys319Tyr) (3/42 alleles; 7.14%). The homozygous variants corresponded to 11 patients, seven cases for c.2299del (p.Glu767Serfs*21) and one for each of the follow: c.2276G > T (p.Cys759Phe); c.12067-2A > G; c.486-14G > A (Intronic); c.5278del (p.Asp1760Metfs*10); c.2276G > T (p.Cys759Phe). In the homozygous cases, endogamy or consanguinity was positive in 3/11 and 1/11 was an isodisomy of chromosome 1. For the simplex RP cases, the most prevalent allele was c.2276G > T (p.Cys759Phe (5/12 alleles), only one case with this variant was in homozygous state. The remaining, a CRD case was a compound heterozygote. Consanguinity or endogamy was denied for RP and CRD.

### Partially solved cases

A total of 10/126 (7.9%) was classified as partially solved. The prevalence of the pathogenic or probably pathogenic variants were distributed in heterozygous state as follows: *USH2A* 4/10, *KIZ* 2/10, *ABCA4* 1/10, *MFRP* 1/10, *CRB1* 1/10, *CLN5* 1/10. None of these variants were novel.

### Suspected causal VUS and unsolved cases

In this cohort, 17.46% (22/126) cases remain unsolved, their clinical diagnoses were 13 RP, 5 CRD, 1 LCA, 1 BD, 1 XLR, 1 Usher syndrome. Unclassified genotypes were due to the identification of only one recessive pathogenic variant without clinical correlation or only VUS. Three of these cases had relevant molecular findings. A 49 years female with nyctalopia since age 3, followed by peripheral vision loss. At 40 years, bone spicules were found, and RP was diagnosed. Two VUS on opposite chromosomes were identified in *CNGB1* c.1676C > A (p.Thr559Lys) and c.1720C > T (p.Leu574Phe). Considering her clinical presentation and the possible effects on the protein, these variants could be causal. A second case is a 58 years male, who started with photophobia at 42 years followed by dyschromatopsia. He carries an heterozygous VUS in *GUCY2D* c.2795 T > G (p.Met932Arg). Considering his clinical phenotype, the mother visual deficiency, and predictions on the effect of this variant on protein structure and function [85], we assume that this variant is likely disruptive. The third case is a 17 years male patient with X-linked retinoschisis. Since he was 6 years he presented central blurry vision. Glasses were prescribed but they did not improve his vision. At 14 years, a retinologist noticed foveal schisis, and asked for optical coherence tomography (OCT) which supported this diagnosis. In his molecular test a VUS in hemizygous state was found in *RSI* c.341C > T (p.Ser114Phe). These three unsolved cases were isolated cases, the four VUS were novel. The remaining cases didn’t have a clinical correlation with the encountered VUS.

## Discussion

Genetic variants for IRDs are present in up to 36% of the world population, when accounting for asymptomatic carriers of recessive mutations [4]. As many of these mutations could be novel in nature and geographically prevalent due to founder effects, the genetic study of IRDs in diverse groups of populations is highly relevant [13]. The enormous genetic and phenotypical heterogeneity of IRDs is reflected in this work. The cohort contains 126 cases, pathogenic or probably pathogenic variants were identified in 94 cases, 10 cases were partially solved cases and 22 persisted as unsolved cases. To the authors’ best knowledge, two previous large cohorts have reported genetic findings in IRDs in patients originating from central and south Mexico [13, 14], so it is important to complete the information for these retinal pathologies in other regions of the country. In addition, it is also important to consider the genetic differences in the northeastern population which could possess a greater proportion of European alleles [86, 87] compared with the central/south Mexican populations.

A similar number of patients were examined across the previous cohorts and the present study (144, 143 and 126 patients respectively) [13, 14]. Regarding gender distribution, while a slight female predilection was found in the present study (56.3 vs 44%), the opposite was reported by Villanueva*, *et al*.* [13] (58.3 vs 41.7% of males and females respectively) and no gender distribution was reported by Zenteno*, *et al*.* [14]. A comparison of the characteristics of the patients from the three cohorts is shown in Table 2. The most common, pre-sequencing diagnosis was RP across all three cohorts. In addition, the mutation detection rate was similar in all 3 studies, ranging from 70–80% and the most detected mutation type were missense variants across all three studies. On the other hand, the most frequently encountered affected gene in the present study was *USH2A* (29.78%). This number differs from previous studies on Mexicans, whose reports were 3.5% [13] and 7% [14]. In the other cohorts *ABCA4* was more frequently altered [13, 14]. Finally, the proportion of unsolved cases was similar between the present study and Villanueva-Mendoza*, *et al*.* [13] (15.3 vs 17.46%) and higher in the cohort from Zenteno*, *et al*.* [14] (33.5%). The considerable proportion of unsolved cases could be related to gene panel limitations, including its capability to detect *RPGR* variants, CNVs, and intronic variants.

The most frequent pathogenic variant of the whole cohort was c.2299del (p.Glu767Serfs*21) in *USH2A*. This variant is in exon 13 is the subject of a phase 3 therapy clinical trial involving the investigational new drug Ultevursen, an antisense RNA oligonucleotide (NCT05158296). The high prevalence of the c.2299del variant in *USH2A* found in the present study could be relevant for this therapy if it is approved. There is sufficient clinical evidence that the c.2299del (p.Glu767SerfsTer21) variant is pathogenic and highly prevalent. A recent report on the frequency of this variant in the cases from central and southern Mexico accounts for 7 and 23% of the alleles causing non-syndromic RP and Usher syndrome, respectively [88]. Furthermore, the Genome Aggregation Database v.4.0.0 shows that the frequency of this allele in the admixed Latino population is 0.0014, the highest globally, followed by the 0.001176 frequency in non-Finnish Europeans [89]. Dreyer*, *et al*.* reported the c.2299del variant in patients from Europe, North and South America, South Africa, and China and noted that it is associated to a core haplotype suggesting that this mutation is an ancestral mutation spread in Europe and introduced in the Americas after the conquest [90].

Other genetic therapies in development are relevant for this report. The vMCO-010 in phase 2 clinical trial (NCT05417126) and rAAV2tYF-GRK1-RPGR in phase 1/2 clinical trial (NCT03316560) are two promising therapies for patients with STGD/MD and X-Linked RPGR, respectively. On the other hand, no patients with RPE65 variants were found in this cohort, therefore no candidates for the only approved gene therapy for IRDs are reported.

Of all causative variants in this cohort, 14 were novel. Eight of these were missense variants (one pathogenic, five VUS, and two probably pathogenic). Three were CNVs, all classified as pathogenic, two frameshift variants classified as VUS and one splicing classified as probably pathogenic. All VUS are suggested to be disrupting variants but there was not enough evidence to classify them as pathogenic. The three novel CNVs may be explained by the recent developments in NGS detection by NGS suggesting that CNV detection will improve the diagnosis rate.

There were some interesting cases. The first was a case with isodisomy of chromosome 1, which has been already reported [91]. We also detected a patient with type IIIC mucopolysaccharidosis (Sanfilippo C), a 55 years patient, with severe intellectual disability, speech impairment, deafness, coarse facies, motor deterioration and late onset RP. He had an affected sister who suddenly died at 21. This patient has two novel *HGSNAT* variants, one CNV classified as pathogenic, and one missense classified as VUS. This could be the first case reported in a Mexican patient. Another interesting case was a woman patient with Arts syndrome, an X-linked disorder, who suffers from retinal dystrophy, optical atrophy, deafness, short stature, and intellectual disability. Among the unsolved cases, there is one case with isolated RP and two biallelic variants in *CNBG1*, c.1676C > A (p.Thr559Lys) and c.1720C > T (p.Leu574Phe), both classified as VUS. Looking into the clinical presentation of this patient and the changes at the protein level, we can conclude that there is a clinical correlation and that those VUS are probably disruptive. Another remarkable case was a 15 years male with juvenile retinoschisis, carrying a VUS in hemizygous state at *RS1* identified as c.341C > T (p.Ser114Phe). This variant is highly likely to be the cause of the clinical presentation. Another notable case, was a 58 years male with CRD, with the VUS c.2795 T > G (p.Met932Arg) in heterozygous genotype in *GUCY2D*. The clinical presentation, the suggestive mother visual symptoms, and the amino acid changes suggest that this variant is highly suspicious of being disruptive as suggested by algorithmic predictions [92].

This study has some limitations, such as the sample size, as there were only 126 patients. Another limitation could be that this panel only encompasses genes in nuclear DNA and very few intronic variants. Sequence changes in the promoter, non-coding exons, and other non-coding regions were not covered. Additionally, no ancestry and founder effect studies were performed.

## Conclusions

This study provides more information about the landscape of the mutations in the IRDs patients in Mexico. Contrary to previous studies in other locations in Mexico, *USH2A* was the most frequently affected gene in the present study. This suggests that there are differences in the genetic component of IRDs between the various regions of the country. It is paramount to study other regions that had not been studied yet, and to create a national registry of IRDs patients. Therapies may arrive soon, or there could be some protocols carried out in Mexico, there, lies the importance of an accurate diagnosis in these patients.

### Supplementary Information

**Additional file 1: Supplementary Table 1.** Demographic and clinical characteristics of study participants.

## Data Availability

The authors have provided a normal table and a supplementary material with all the genetic and clinical results of the present paper. Additionally, all the data from the present manuscript is available in the database LOVD v.3.0. The specific reference is available in the results section. Further data availability can be asked for by contacting the corresponding author. Database link: https://databases.lovd.nl/shared/variants#order=VariantOnGenome%2FDNA%2CASC&search_VariantOnGenome/Reference=Cruz%20RA%2C%20et%20al.%2C%202023&page_size=100&page=1.
